# Irreversible electroporation promotes a pro-inflammatory tumor microenvironment and anti-tumor immunity in a mouse pancreatic cancer model

**DOI:** 10.3389/fimmu.2024.1352821

**Published:** 2024-04-22

**Authors:** Khan Mohammad Imran, Rebecca M. Brock, Natalie Beitel-White, Manali Powar, Katie Orr, Kenneth N. Aycock, Nastaran Alinezhadbalalami, Zaid S. Salameh, Paige Eversole, Benjamin Tintera, Justin Markov Madanick, Alissa Hendricks-Wenger, Sheryl Coutermarsh-Ott, Rafael V. Davalos, Irving C. Allen

**Affiliations:** ^1^ Medicine and Health, Virginia Polytechnic Institute and State University, Roanoke, VA, United States; ^2^ Department of Electrical and Computer Engineering, Virginia Polytechnic Institute and State University, Blacksburg, VA, United States; ^3^ Department of Biomedical Engineering and Mechanics, Virginia Polytechnic Institute and State University, Blacksburg, VA, United States; ^4^ Department of Biomedical Sciences and Pathobiology, Virginia-Maryland College of Veterinary Medicine, Blacksburg, VA, United States; ^5^ Virginia Polytechnic Institute and State University, Blacksburg, VA, United States; ^6^ Department of Surgery, Carilion Clinic and Virginia Tech Carilion School of Medicine, Roanoke, VA, United States; ^7^ Department of Basic Science Education, Virginia Tech Carilion School of Medicine, Roanoke, VA, United States

**Keywords:** ire, tumor ablation, immunomodulation, tumor recurrence, cell cycle arrest, tumor microenvironment

## Abstract

Pancreatic cancer is a significant cause of cancer-related mortality and often presents with limited treatment options. Pancreatic tumors are also notorious for their immunosuppressive microenvironment. Irreversible electroporation (IRE) is a non-thermal tumor ablation modality that employs high-voltage microsecond pulses to transiently permeabilize cell membranes, ultimately inducing cell death. However, the understanding of IRE’s impact beyond the initiation of focal cell death in tumor tissue remains limited. In this study, we demonstrate that IRE triggers a unique mix of cell death pathways and orchestrates a shift in the local tumor microenvironment driven, in part, by reducing the myeloid-derived suppressor cell (MDSC) and regulatory T cell populations and increasing cytotoxic T lymphocytes and neutrophils. We further show that IRE drives induce cell cycle arrest at the G0/G1 phase *in vitro* and promote inflammatory cell death pathways consistent with pyroptosis and programmed necrosis *in vivo*. IRE-treated mice exhibited a substantial extension in progression-free survival. However, within a span of 14 days, the tumor immune cell populations reverted to their pre-treatment composition, which resulted in an attenuation of the systemic immune response targeting contralateral tumors and ultimately resulting in tumor regrowth. Mechanistically, we show that IRE augments IFN- γ signaling, resulting in the up-regulation of the PD-L1 checkpoint in pancreatic cancer cells. Together, these findings shed light on potential mechanisms of tumor regrowth following IRE treatment and offer insights into co-therapeutic targets to improve treatment strategies.

## 1 Introduction

Overall cancer death rates are declining continuously with the emergence of new treatment paradigms. However, the mortality rate of pancreatic cancer remains unacceptably high. For pancreatic cancer patients, surgery remains the only curative therapy, but due to the localization of the tumor within a complex organ system and its proximity to nearby critical structures, most patients are ineligible to receive surgical resection (1). Furthermore, pancreatic cancer is highly metastatic, notoriously immunosuppressive, and typically becomes chemo-resistant (1). Traditional treatment options, such as chemotherapy, normally extend life expectancy by just several weeks (2), whereas radiation therapy has shown no increase in life expectancy (3). Even the most impressive emerging immunotherapeutic have proven limited in treating pancreatic cancer due to the immunologically “cold” microenvironment that inhibits the pro-inflammatory, anti-tumor immune cells (4). Thus, new therapeutic paradigms are direly needed.

The use of minimally invasive, focal tumor ablation modalities to treat pancreatic cancer is currently an area of emerging potential. These modalities utilize electrical, ionizing, thermal, or mechanical forces to ablate targeted tumors with a high level of precision. However, in the context of pancreatic cancer, the utilization of thermal or ionizing approaches has significant limitations due to the risk of damaging vital structures. These approaches also suffer from the heat sink effect, which can result in a high degree of variability in the ablation zone and the potential risk of thermal injury-stimulated pancreatitis (5). Electroporation-based technologies have the potential to overcome many of the challenges currently faced in treating pancreatic cancer. Specifically, irreversible electroporation (IRE) has shown promise in both the clinic and in pre-clinical studies as a locoregional therapy for the treatment of other types of cancer, including liver and breast (6–9). IRE delivers short, unipolar electric pulses that are largely non-thermal and alter the cell transmembrane potential to promote cell death (10, 11). In clinical trials, the induction of tumor cell death lowers the overall tumor burden and enhances progression-free survival in patients (9, 12, 13). However, this expanded progression-free survival appears to be limited in some patients, resulting in studies that explore combination therapeutic approaches to optimize and improve patient outcomes (9, 14, 15).

The induction of a robust, systemic anti-tumor immune response has been found to be a major advantage of IRE and other electroporation based therapeutic approaches, albeit the mechanism of these effects remains highly enigmatic (8, 16, 17). Previous studies conducted by our research team and others have found that IRE treatment of mammary cancer cells significantly alter the tumor microenvironment, shifting the immunologically “cold” tumor towards a more pro-inflammatory, anti-tumor “hot” microenvironment (8, 18). This was in part associated with a reduction in immunosuppressive cell populations, the release of damage associated molecular patterns that enhance the innate immune response, and the production of pro-inflammatory mediators and cytokines (8, 18–20). Importantly, the enhanced activation of the innate immune system, facilitated a more robust adaptive immune response, resulting in augmented systemic anti-tumor immunity and ultimately reduced metastatic lesions (21–23).

The application of IRE has been nearly equal for pancreatic and prostate cancer in clinics, yet it has been less frequently utilized for breast, and kidney cancers (6). Despite this, the mechanisms behind cell death and tumor recurrence in pancreatic cancer remain poorly understood. Most of the studies to date have focused on the type of cell death initiated following treatment and local innate immune responses (9, 24, 25). We have previously hypothesized that IRE treatment has the ability to cause release of DAMPs and neoantigens, which has the potential to mitigate the local immunosuppressive factors and enhance the activation of an anti-tumor immune response (19). Here, we confirm this postulate and show that IRE stimulates pro-inflammatory cell death in pancreatic tumors, while significantly altering the tumor microenvironment. This is driven, in part, by an increase in anti-tumor immune cell populations resulting in an overall improvement in tumor progression and clinical outcomes. However, in the context of a partial tumor ablation, these effects are temporary, with the treated tumors regrowing. This is characterized by a reversion back to a localized immunosuppressive tumor microenvironment, which makes partial ablation model a great tool to study mechanism of tumor regrowth. Our results suggest that the generation of IFN-γ and subsequent upregulation of PD-L1 in the pancreatic cancer cells following IRE is highly associated with this regrowth phenotype. Intriguingly, this mechanism appears to be specific for the pancreatic cancer cells evaluated in this study and does not occur in other cancer cell types that generate a more robust abscopal effect following IRE-based therapeutic applications. Together, these findings may shed light on the often-conflicting findings related to the abscopal effect and also serve to emphasize the importance of further research to optimize IRE-based therapeutic approaches.

## 2 Materials and methods

### 2.1 *In vitro* IRE treatments

The Pan02 murine pancreatic adenocarcinoma cell line utilized in this study was originally obtained from the National Cancer Institute (NCI) Division of Cancer Treatment and Diagnosis Tumor Repository and cultured according to their recommended protocols. Specifically, we maintained Pan02 cells in Roswell Park Memorial Institute (RPMI) 1640 medium (ATCC) supplemented with 10% fetal bovine serum (R&D Systems) and 1% penicillin-streptomycin (Gibco). Cells were sub-cultured up to 16 passages and subjected to routine mycoplasma testing, which showed negative results. These cells were harvested with 0.25% trypsin (ThermoFisher) suspended in a low-conductivity sucrose solution (85 g sucrose, 3.0 g glucose, 7.25 ml RPMI, and 992.75 ml DI water), as described in a previous study (8), at a concentration of 6x10^6^ cells per milliliter. Subsequently, the Pan02 cells were subjected to IRE treatment within 4mm electroporation cuvettes. The treatment process was conducted in a safety stand using an irreversible electroporation generator (Harvard Apparatus) and a fiber optic temperature probe (Advanced Energy). This treatment consisted of 4 sets of 25 pulses, each with a duration of 100 µs and a 30-second delay between sets and different voltage (100V-1200 V) as specified in the figure legend. Following treatment, the cells were allowed to rest for 5 minutes on ice. Next, samples were collected to assess cell viability using Trypan Blue (ThermoFisher). The treated cells were then divided into three wells at a density of 1x10^6^ cells per well and diluted with complete growth media at a 1:4 ratio. Cell samples were harvested at 8-, 24-, and 48-hours post-treatment. The supernatant from these samples was collected for lactate dehydrogenase (LDH) assessment (ThermoFisher Pierce). The cell pellets were processed for RNA extraction using TRIzol (Sigma Aldrich) or for protein extraction in RIPA cell lysis buffer collection solution, which contained 1x protease inhibitor (Fisher Scientific). Each time point was evaluated in triplicate for each electric field strength, and this entire process was biologically replicated three times, resulting in a total of n=9 for each experimental group. Furthermore, cell cycle analysis using propidium iodide DNA staining (Abcam) was performed according to the manufacture protocol and analyzed by flow cytometry.

### 2.2 Experimental animals

All experimental procedures were performed following Institutional Animal Care and Use Committee (IACUC) approval and by following the guidelines outlined in the National Institutes of Health (NIH) Guide for the Care and Use of Laboratory Animals. The animals were housed in a controlled environment in specific pathogens free (SPF) conditions, maintaining 12-hour light/dark cycles at a temperature range of 20–24°C with a relative humidity of 55% ± 10%. They were provided with unrestricted access to food and water. To start the experiments, male and female C57Bl/6J mice (JAX® Mice Strain), aged between 8 and 12 weeks, were subcutaneously injected with 6x10^6^ Pan02 cells suspended in Matrigel (Corning) on the right flank. The use of Matrigel helped to facilitate the formation of nodular subcutaneous tumors. Throughout the course of the study, the health condition of the mice and the size of the tumors were closely monitored, with measurements performed three times a week or more frequently, contingent on the animal’s clinical progression and tumor burden. Tumor size was determined using Vernier calipers to measure the square root of the product of two perpendicular diameter measurements, following a methodology established in prior research (8). Mice were euthanized at designated experimental timepoints or at the conclusion of the study, either when tumors reached a diameter of 1.6 cm or when the mice displayed clinical signs of distress.

### 2.3 *In vivo* IRE application

Mice were anesthetized with an isoflurane (Vet One) inhalant via nose-cone during treatment. Paired needle electrodes (~21 gauge) spaced 4 mm apart were inserted into the tumor in four separate directions sequentially (See **Supplementary Figure 1**) and 800 V was applied with an irreversible electroporation generator (Harvard Apparatus). Treatments were applied when the tumor reached around 0.5 cm in diameter, which was 9-12 days after injection. The application was for four sets of 100 µs (25 pulses a set, one set per direction, a total of 100 pulses) to ensure tumor coverage and mimic clinical application. Control mice were anesthetized in the same manner with electrodes inserted in a similar fashion, without an applied electrical current. All animals recovered from the IRE treatment.

### 2.4 Tissue collection and histopathology

Mice were euthanized, whole blood was collected by intracardiac needle puncture, and the tumor was excised. For histopathology, tumors were processed to allow for maximum tumor margin observation. The thoracic cavity was opened, the heart perfused with phosphate-buffered saline (ThermoFisher), and lungs removed for metastasis evaluation. In the subcutaneous model, the lungs are a common location to assess for metastatic lesions. The lungs were excised, inflated with 10% formalin, and fixed in cassettes in 10% formalin. Fixed tissues were embedded in paraffin, sliced in 5µm sections, and mounted on slides. Slides were stained with H&E and blind graded by a board-certified veterinary pathologist (SCO) for the extent of necrotic tissue in the primary tumor and the number of metastatic lesions identified per lung. Additional tumor specimens and lung tissue were snap-frozen on alcohol slurry for later RNA and protein analysis.

### 2.5 Gene expression analysis

Gene expression and IPA analysis was conducted as previously described by the research team (8). Briefly, Pan02 tumor tissue RNA was isolated via RNeasy (Qiagen) and quantified using a Nanodrop 2000 (Thermofisher). Samples were equally pooled in each group for cDNA generation via RT2 First Strand (Qiagen). Gene expression was determined using an RT^2^ profiler array PAMM-033Z and PAMM-052Z (Qiagen) on an ABI 7500 Fast Block. A total of 171 unique genes were assessed. PCR results were analyzed using the Qiagen Data Analysis browser service to normalize data and calculate fold regulation based on array housekeepers. Individual sample gene expression was verified and validated with TaqMan primer probes (Thermo scientific) for specific, targeted genes. Gene expression data from RT2 Profiler arrays were analyzed using Ingenuity Pathway Analysis software (IPA, Qiagen). IPA utilizes fold regulation of gene expression and publicly available databases to predict changes to biological pathways. Z-scores are calculated by the IPA software to determine the predicted upregulation or downregulation of a pathway based on the number and strength of gene expression changes found to be involved.

### 2.6 Protein analysis

For the timepoint study, tumors were collected from IRE treated and untreated mice (7 mice per group) at 1, 3, 6, 9, 14, 21, and 28 days post-treatment, samples were pooled for western blot analysis. Blood sera was isolated from whole blood and assessed via enzyme-linked immunosorbent assay (ELISA) for a range of inflammatory mediators, including IFN-γ. U251 and 4T1 cells were obtained from ATCC and cultured according to ATCC cell culture protocols. The osteosarcoma (DLM8) cell was generously provided by Dr. E. Kleinerman (M.D. Anderson Cancer Center). To maintain DLM8 cells, DMEM with GlutaMAX (Thermo Fisher Scientific) was used, supplemented with 10% FBS (Atlas), 1 mM sodium pyruvate (Thermo Fisher Scientific), 1% nonessential amino acid solution (Thermo Fisher Scientific), and 1% penicillin/streptomycin (Thermo Fisher Scientific). Subculturing was performed at ~80% confluency using 1% trypsin supplemented with 0.5 mM EDTA (Thermo Fisher Scientific). Cells were treated with cytokines as indicated in the figure legend for 24 hours. For protein collection, cells were washed twice with ice-cold 1 × PBS and subsequently lysed in RIPA lysis buffer. This lysis buffer was supplemented with a protease and phosphatase inhibitor cocktail (ThermoFisher). The lysates were then harvested through scraping, followed by incubation on ice for 15 minutes. Tumor tissues collected after euthanasia were minced in RIPA lysis buffer followed by ultrasonication on ice. Subsequently, specimens were centrifuged at 14,000 × g for 15 minutes at 4°C to collect the supernatant. The protein concentration was determined using a BCA Protein Assay Kit (Thermo Fisher). Equal amounts of protein (10 µg) were loaded for each sample and separated on a 4–12% sodium dodecyl sulfate polyacrylamide gradient gel (NuPAGE™ 4 to 12%, Bis-Tris, 1.0–1.5 mm, Mini Protein Gels, (ThermoFisher) at 165 V for 45 minutes. Following electrophoretic separation, the proteins were transferred onto a polyvinylidene difluoride (PVDF) membrane using an iBlot™ 2 Gel Transfer Device (ThermoFisher) at 13 V for 7 minutes. Subsequently, the membranes were blocked with 5% BSA in 1 × Tris-buffered saline (TBS) containing 0.1% Tween-20 for 1 hour at room temperature while on a shaker. Next, specimens were incubated with primary antibodies overnight at 4°C. The membranes were then washed three times for approximately 10 minutes at room temperature. The PVDF membrane was subsequently subjected to agitation on a shaker with a specific horseradish peroxidase-conjugated secondary antibody for 1.5 hours at room temperature, followed by three washes. Immunoreactive protein signals were detected using a chemiluminescent ECL assay. Western blotting was performed for p-STAT3 (Cell Signaling Technologies Cat#9145T), p-STAT1(Cell Signaling Technologies Cat#9167T), β-actin (Cell Signaling Technologies Cat#4970T), and PD- L1(Abcam, Cat#ab205921). Blots were assessed with a Licor Odyssey XF (LI-COR) and normalized to β-actin.

### 2.7 Flow cytometry

Pan02 tumor tissue was harvested, mechanically digested, and cells diluted in complete RPMI 1640 (ATCC). Cells were fixed with fixation buffer (eBiosciences) and stored in 4 °C. For cell surface staining, cells were incubated with anti-CD16/32 (Fc block, ThermoFisher Cat#553142) in FACS buffer (ThermoFisher) followed by staining for 30 minutes in the dark with desired antibodies. Cells were then permeabilized using the True-Nuclear Transcription Factor Buffer Set (Biolegend), following manufacturer’s guidelines for use with FoxP3 (ThermoFisher) binding. Cell staining and population identification can be found in **Supplementary Table 1**. Cell populations were identified with BD FACS Aria Fusion flow cytometer (BD Biosciences). Downstream analysis was performed with FlowJo.

### 2.8 Contralateral tumor study

Subcutaneous pancreatic tumors were introduced by implanting 6x10^6^ Pan02 cells in the left and right flank of 6- to 8-week-old female C57BL/6J mice. IRE was performed when tumors reached ~0.5 cm diameter (9-12 days after injection) using an irreversible electroporation generator (Harvard Apparatus) with a pair of 21-gauge needle probe, separated by 4 mm, to deliver a total of 100 pulses at 800 V on the right flank tumor (only) as described earlier.

### 2.9 Statistical analysis

The data analysis was performed using GraphPad Prism, version 9. To compare two experimental groups, a two-tailed Student’s t-test was employed. Data involving multiple comparisons, one-way and two-way ANOVA tests were applied as deemed appropriate, followed by Mann-Whitney or Tukey post-tests for conducting multiple pairwise comparisons. Statistical significance was defined as a p-value of 0.05 or less. All data are presented as the mean value with the associated standard error of the mean (SEM).

## 3 Results

### 3.1 IRE induces cell cycle arrest and cell death

**Figure 1:**
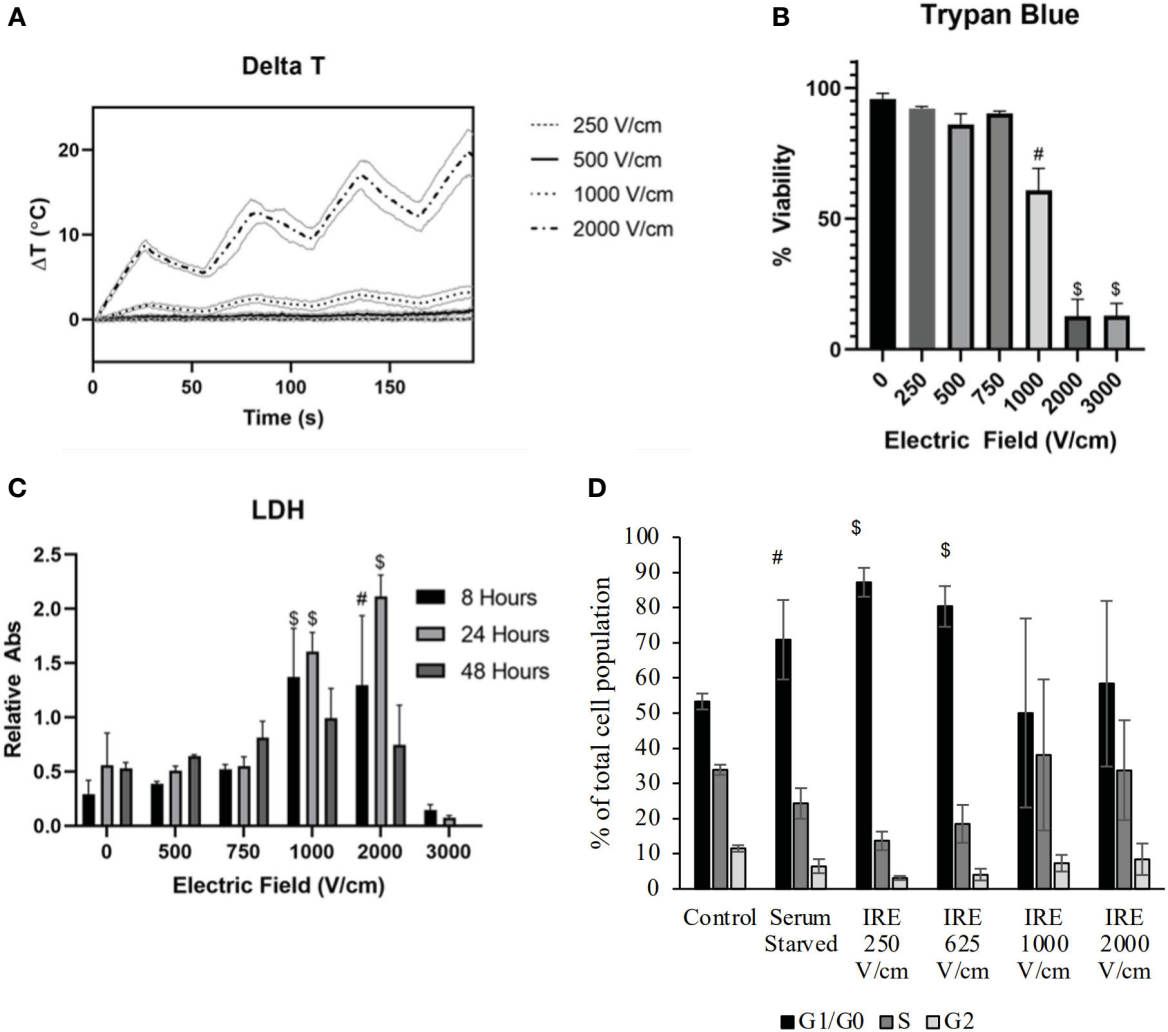
IRE induces pancreatic cancer cell death through the induction of cell cycle arrest. Murine pancreatic Pan02 cells were treated in suspension with IRE at various electric field strengths. **(A)** Temperature was measured via fiberoptic probe throughout treatment. n=11, SD shown. **(B)** Trypan Blue exclusion was used to measure cell death after IRE application. **(C)** LDH release into the supernatant was measured at various timepoints as an indicator of cell death over time. n=9, combination of three biological replicates, SEM shown. P-value< #<0.001, $<0.0001. **(D)** Cell cycle arrest was evaluated utilizing flow cytometry after staining Pan02 cells with propidium iodide 24-hour post-IRE treatment.

It has already been established that IRE is effective at ablating cells. Indeed, cell death appears to occur along a continuum based on energy exposure and distance from the electrodes, with necrosis occurring closest to the electrodes, extending out to zones of necroptosis, pyroptosis, and apoptosis, eventually forming a zone of reversible electroporation of cells at the treatment margin (26–30). However, these processes have not been fully defined in the context of pancreatic cancer cells and cell lines. To evaluate cell death processes in the Pan02 murine pancreatic cancer model, we initially treated cells with variable IRE electric fields over a time course and investigated cell death mechanisms (**Figure 1**). IRE treatment ***in vitro*** indicated little to no increase in temperature throughout the application until reaching 2000 V/cm, which represented the higher end of clinical application in terms of amperage (**Figure 1A**). As the electric field increased, we observed a significant decrease in cell viability at field strengths greater than 1000 V/cm (**Figure 1B**). Consistent with the cell death findings, we also observed increased markers of cell death, including increased LDH levels over time following IRE application using electric fields between 1000 and 2000 V/cm (**Figure 1C**). LDH is a common marker of cell death and is generated when the plasma membrane is damaged. We observed LDH levels peak at 24 hours post-IRE treatment and rapidly decline by 48 hours (**Figure 1C**), suggesting that cells underwent delayed and prolonged programmed cell death. It should also be noted that the levels of LDH were minimal at 3000 V/cm at all timepoints evaluated (**Figure 1C**). This is due to the immediate death of cells at the higher electric field associated with thermal damage rather than electroporation. Cell death is a highly nuanced biological process. To better define the underlying mechanism driving the observed cell death, we next evaluated Pan02 cell cycle progression 24 hours following IRE treatment (**Figure 1D**; **Supplementary Figure 2**). At lower electric fields, we observed that IRE induced cell cycle arrest at the G1/G0 phase at ranges between 250 – 625 V/cm (**Figure 1D**). Higher electric fields increased variability but showed a trending increase in cells in both G1/G0 and S phase (**Figure 1D**). Cell cycle arrest in the G1/G0 phase is anticipated to facilitate apoptosis, whereas the higher electric fields are anticipated to promote the activation of multiple cell death pathways that would be consistent with our combined data.

### 3.2 IRE induces proinflammatory cell death and limits disease progression *in vivo*

**Figure 2:**
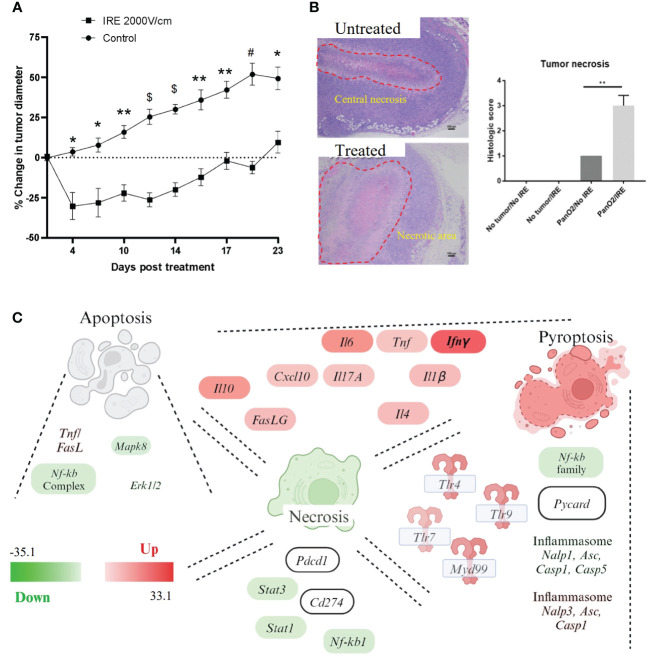
IRE ablates pancreatic tumors and induces proinflammatory programmed cell death *in vivo*. Pan02 cells were injected subcutaneously in immunocompetent mice and treated with IRE once they reached 0.5cm in size. Tumor tissue was evaluated for tissue necrosis and cell death 24 hour post-treatment. **(A)** Tumor diameter post-treatment showed a significant decrease of around 30% post-IRE treatment and a 12-day delay in tumor growth compared to untreated control tumors. **(B)** H&E staining of tumors and grading of necrosis (1=low-grade necrosis, 4= few to no detectable cancer cells). Untreated tumor shows central necrosis (indicated by dotted red line) and treated tumor shows significantly larger necrotic area (dotted red line) with clear treatment margins. n=5, SEM shown. p-value *<0.05, **<0.01, #<0.001, $<0.0001. **(C)** Gene expression profiling was conducted using rtPCR based profiling arrays and pathway analysis was conducted using Ingenuity Pathway Analysis (IPA) software, which identified apoptosis, necrosis, and pyroptosis as being significantly impacted by IRE treatment. Specific genes relevant to each pathway and found to be significantly up- or down-regulated (Scale -33 to +33) following IRE treatment are also indicated in the schematic. Gene expression heatmap data was transposed onto the respective gene/pathway identified in the schematic.

The data discussed above and by others suggests that IRE induces cell death in Pan02 cells over time, with lower electric fields inducing cell cycle arrest and apoptosis, whereas higher fields appear to induce multiple types of cell death in the Pan02 cells. To evaluate this *in vivo*, we engrafted Pan02 cells subcutaneously in wildtype C57Bl6 (WT) mice and monitored tumor progression pre- and post-IRE treatment (**Figure 2**). Tumors were allowed to grow until the tumor reached ~0.5cm in diameter, which typically occurred between days 9-12 days post-engraftment. The subcutaneous engraftment allowed for highly accurate assessments and visualization of tumor progression, coupled with precise IRE treatments that are critical for this stage of proof-of-concept studies with this technology. IRE application mimicked clinical application by being performed with similar parameters in altering pulse sets to ensure coverage of the three-dimensional shape of each tumor and a 30- second delay between sets to dissipate the effects of Joule heating. Within the first 5 days post-IRE treatment, we observed a significant reduction in Pan02 tumor size compared to sham treated tumors in control animals (**Figure 2A**). Tumor progression was effectively stalled until day 10, when tumor progression gradually accelerated, eventually reaching the initial starting size of 0.5 cm on or about day 20 post-treatment (**Figure 2A**). We used a partial tumor ablation model, which allows for robust assessments of the changes in the tumor microenvironment and tumor regrowth following treatment. *In vitro*, treatment with 2000 V/cm resulted in 70-80% cell death (**Figure 1B**). *In vivo* the tumor size reduced by 20% to 55% (**Figure 2A**). As expected based on the IRE parameters utilized, none of the tumors showed complete regression (**Figure 2A**). Histopathology assessments of the ablation zone following treatment demonstrated successful tumor ablation, with a significant increase in the size of the necrotic region of the tumor that ranged from 50% to 70% of the total mass (**Figure 2B**, marked with red line). Pan02 tumors typically have a zone of necrosis in their core (untreated; **Figure 2B**). However, this was significantly enlarged following IRE treatment and a large region of acellular debris was observed in the treatment zone (treated; **Figure 2B**). These findings were confirmed by histopathology scoring by a blinded, board-certified veterinary pathologist with experience evaluating tumor necrosis/ablation effects (S.C.O.) using scale 0-4 where “0” no necrosis and “4” 100% necrosis (**Figure 2B**, right panel). In summary, treatment with IRE was highly effective at ablating the Pan02 tumor; however, regrowth over time appears to be a significant concern.

To better define the mechanism of tumor ablation and identify the relevant cell death pathways activated by IRE ablation *in vivo*, we next profiled gene expression using rtPCR based pathway focused arrays targeting cancer and immune system relevant mechanisms (**Figure 2C**). Gene expression was profiled 24 hours post-IRE treatment in either the treated or sham treated Pan02 tumors (**Figure 2C**). Expression data was analyzed using Ingenuity Pathway Analysis (IPA) and key genes and pathways that were either up- or down-regulated were identified and illustrated in the schematic shown in **Figure 2C**. Our gene expression data analysis reveals pathways associated with apoptosis, necrosis, and pyroptosis are impacted by IRE treatment. In the context of apoptosis, cell death was associated with an increase in TNF and FasL signaling and a decrease in signaling associated with Mapk8 and Erk1/2 (**Figure 2C**). Conversely, pathways associated with necrosis signaling were generally down-regulated (**Figure 2C**). However, a specific repertoire of cytokines and inflammatory mediators were found significantly up-regulated, including IL-1β, TNF, IL-4, IL-10, and IL-6 (**Figure 2C**). TLR signaling was also highly up-regulated (**Figure 2C**). This is consistent with other studies following IRE-based ablation in other tumor models and is consistent with the generation of damage associated molecular pattern signaling and innate immune system signaling following local tissue ablation (6, 8, 31, 32). Consistent with the strong IL-1β signal and TLR signaling, we also observed an increase in NLRP3 inflammasome signaling, including signatures consistent with increased NLRP3 and Casp1 (**Figure 2C**). This up-regulation appears to be associated with the NLRP3 inflammasome, as signaling associated with other NLRs, specifically the NLRP1 inflammasome, was down-regulated (**Figure 2C**). Intriguingly, across all cell death pathways, canonical NF-κB signaling was generally decreased (**Figure 2C**), which may explain the limited number of cytokines and inflammatory mediators up-regulated. Each of the inflammatory mediators up-regulated following IRE treatment have multiple promoters that are capable for functioning independently of canonical NF-κB. Also, of relevance to the data presented later in this study, INFγ was the most up-regulated gene in the panel following IRE treatment (**Figure 2C**). Together, these gene expression data identify several pathways associated with apoptosis, necrosis, and pyroptosis that are up-regulated 24 hours post-IRE in pancreatic cancer models and provides insight into the Pan02 microenvironment.

**Figure 3:**
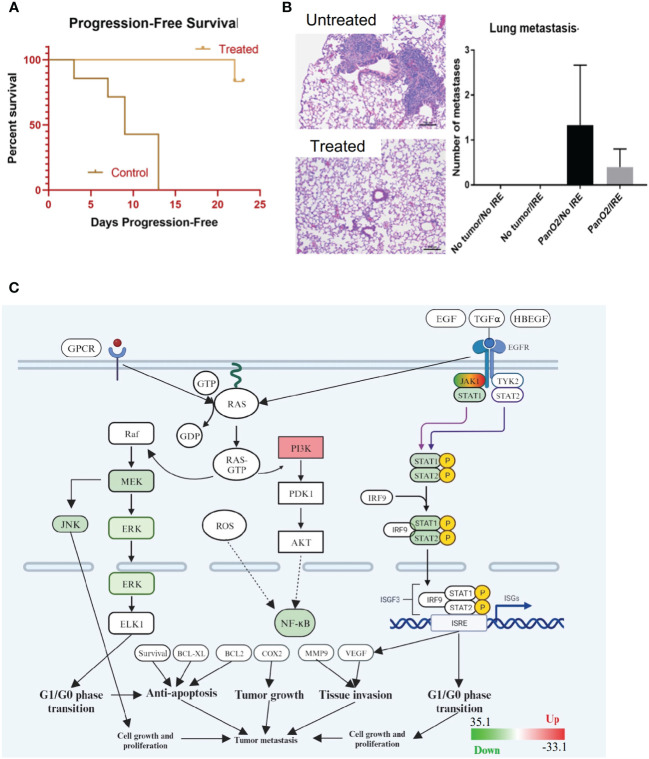
IRE increases progression-free survival through moderately decreasing metastatic burden and down-regulating critical signaling pathways associated with pancreatic cancer. **(A)** IRE-treated mice experienced significantly extended progression-free survival. n=7, p-value=0.0014. **(B)** H&E staining and counting of lung metastases by the end of study (Day 40, 26 days post-IRE treatment) revealed a trending decrease in lung metastatic lesions. n=5. **(C)** Gene expression profiling was conducted using rtPCR based profiling arrays and pathway analysis was conducted using Ingenuity Pathway Analysis (IPA) software. Gene expression data indicated that several genes and pathways associated with cancer biology are dysregulated in the treated tumors following IRA, including JAK/STAT signaling, MEK/ERK, JNK, and NF-κB signaling (Scale -33 to +33).

IRE treatment significantly improved progression-free survival (**Figure 3A**), which is a commonly reported clinical parameter. Previous studies of IRE-based ablation of mammary tumors and other cancer types by our team and others revealed a significant reduction in metastasis to the lungs (8). This was previously shown to be associated with an increase in the systemic anti-tumor immune response and activation of cytotoxic T cells into the tumor microenvironment (8). The sub-cutaneous Pan02 model is considered moderately metastatic and the lung is commonly evaluated (33). Consistent with this characterization of the model, we did identify metastatic lesions in the lungs, of mice engrafted with Pan02 tumors (**Figure 3B**). While the lesions were often large in size when present, animals did not form very many metastases with a large amount of variability per mouse (**Figure 3B**). We did observe a reduction in the number of lesions observed following local IRE treatment of the subcutaneous tumor; however, the effect was not statistically significant (**Figure 3B**). Because this was a partial tumor ablation, it is possible that the reduction in lung lesions may be a result of the primary tumor de-bulking effect of local treatment. An assessment of the gene expression data revealed a significant reduction in a variety of pathways associated with cell cycle regulation, tumor growth, and tissue invasion (**Figure 3C**) following IRE treatment, which are all relevant to tumor progression and metastasis. The gene expression data revealed a significant decrease in JNK and STAT1/2 signaling, MEK/ERK signaling and NF-κB signaling (**Figure 3C**). Only PI3K was consistently found to be significantly upregulated in the context of tumor progression and metastasis signaling following IRE-treatment (**Figure 3C**).

### 3.3 Immune cell populations are temporally altered post-IRE treatment

**Figure 4:**
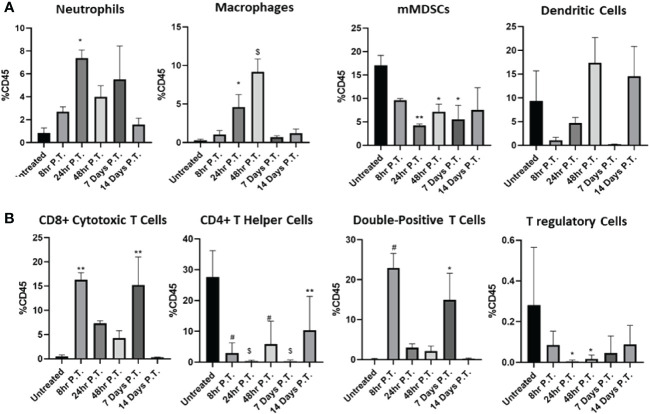
The immune cell populations in the Pan02 tumor microenvironment significantly shift over time post-IRE treatment. Tumors were collected at different points pre- and post-IRE treatment and specific immune cell populations were assessed by flow cytometry. **(A)** Changes in myeloid derived cell populations and **(B)** lymphoid cell populations were quantified over time. Data were analyzed by one-way ANOVA, n=4x3 animals for each collection point, SEM. p-value= *≤0.05, **<0.01, #<0.001, $<0.0001.

Pancreatic tumors create an immunosuppressive tumor microenvironment, driven in part through the recruitment of anti-inflammatory cell types, such as myeloid-derived suppressor cells (MDSCs) (16, 34). To better define the changes in the Pan02 tumor microenvironment, tumors were collected over a 14 day time course, following either IRE or sham treatment and prepared for flow cytometry (**Figure 4**; **Supplementary Table 1**). Representative gating is shown for the untreated and IRE-treated tumors (**Supplementary Figure 3**). Consistent with the increase in DAMP signaling and inflammatory cell death discussed above in **Figure 2C**, within the first 48 hours post-IRE treatment, we observed a significant influx of inflammatory neutrophils, macrophages, and a trending increase in dendritic cells (**Figure 4A**). We also observed a significant influx of CD8+ cytotoxic T cells 8 hours post-IRE (**Figure 4B**), which likely represent the general recruitment of these cells rather than an antigen specific response. The CD8+ T cells reemerged 7 days post-treatment and were completely absent by day 14 post-treatment (**Figure 4B**). A similar pattern was also observed in the CD4+CD8+ double positive T cell population (**Figure 4B**). CD4+ T helper cells significantly decreased following IRE ablation and remained low throughout the time course study, but appeared to trend towards higher percentages over time (**Figure 4B**). The increase in pro-inflammatory immune cells were augmented by a concurrent, statistically significant, decrease in the mMDSC and regulatory T cell populations (**Figures 4A, B**). In sum, most of the changes in cell populations were temporal, with the majority of cell types returning to pre-treatment levels by Day 14 (**Figures 4A, B**).

### 3.4 IRE treatment transiently reduces tumor size in contralateral Pan02 engraftment studies

**Figure 5:**
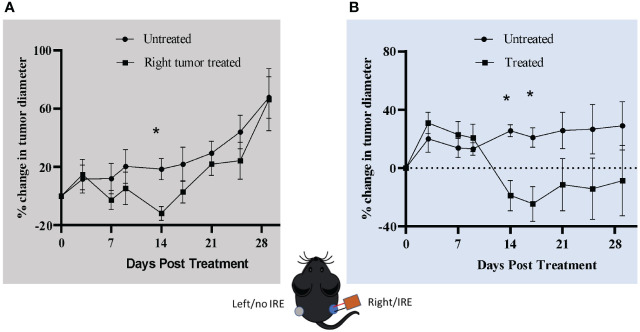
IRE treatment reduces contralateral tumor size. Mice were injected with 6x10^6^ Pan02 cells in both the left and right flank. When the tumor size reached ~0.5 cm in diameter, the right flank tumor was treated with 2000 V/cm IRE. **(A)** Left flank tumor diameter. **(B)** Right flank tumor diameter. Vertical dotted line shows the size on the day of treatment. n=7 for treatment and non-treatment group and n=3 for sham/control group, SEM, p-value**=** *≤0.05.

IRE recruits cytotoxic T-cells in the tumor site, moderately impacts metastases and initiates proinflammatory cell death in the Pan02 tumors. Based on these findings and other studies in a variety of tumor models we would predict that IRE treatment would have a significant effect on the systemic anti-tumor immune response. To evaluate this, we utilized a contralateral tumor engraftment model. We engrafted two tumors, one on each flank of the same C57Bl/6 mice, by injecting 6x10^6^ Pan02 cells. When the tumors reached ~0.5 cm in diameter we treated the right flank tumor with 800 **V** IRE (2 needles, 4 mm gap) and monitored the tumor growth of both tumors. IRE treatment reduced the size of both left and right flank tumors (**Figures 5A, B**). As expected, the tumor size of the treated right flank tumor was significantly reduced compared to the sham treated (untreated) tumors (**Figure 5B**). Consistent with an increase in the systemic anti-tumor immune response, we also saw a significant reduction in the contralateral, left flank tumor in the IRE-treated mice (**Figure 5A**). While this was a statistically significant decrease at the 14 day post-IRE timepoint, the response was only transient. The innate immune response was short lived for the left flank tumor. The range of reduction in tumor size at that time point was 9-23%. None of the tumors showed complete regression. Tumor growth in the contralateral tumor became more aggressive by Day 17, eventually reaching the same size as the contralateral tumor in the sham treated mice by Day 28 (**Figure 5A**). Together, these findings suggest the activation of systemic anti-tumor response following IRE treatment. However, they also point to the presence of a possible negative feedback loop that could potentially result in a more aggressive regrowth.

### 3.5 Robust immune checkpoints are enhanced by IRE treatment in Pan02 cells

**Figure 6:**
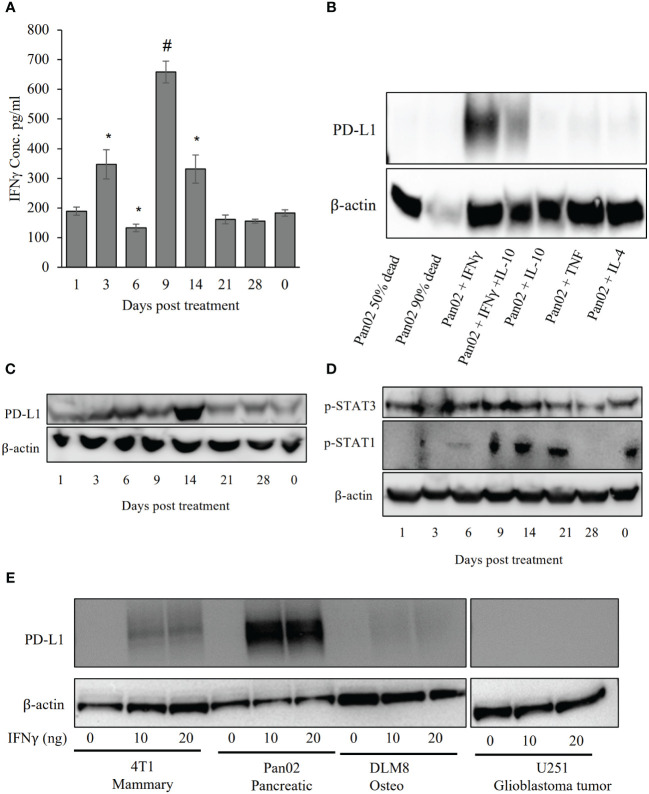
IRE stimulated IFN-γ and PD-L1. **(A)** ELISA for IFN-γ in the blood sera shows an increase in IFN-γ on days 3, 9 and 14 post-IRE. n=7, SEM, p-value= *≤0.05. **(B)** Pan02 cells were treated with 10 ng/ml of selected cytokines and proteins were collected after 24 hour for western blot analysis. *In vitro* IFN-γ treatment shows robust PD-L1 induction. **(C)** Western blot shows peak PD-L1 expression at day 14 post-IRE treatment. **(D)** STAT1, but not STAT3, phosphorylation was found after IRE treatment. **(E)** Mouse Pan02, 4T1, and DLM8 cells were treated with murine recombinant IFN-γ and human U251 cells were treated with human recombinant IFN-γ and PD-L1 levels were evaluated using western blot. n=5, SEM, p-value= *≤0.05, #<0.001.

IRE is highly effective in ablating the primary Pan02 tumor; however, the temporal effects and reversal of the tumor microenvironment are intriguing. To better define the mechanism associated with regrowth we revisited our gene expression data that identified the significant increase in IFN-γ signaling (**Figure 2C**). The PD-L1 checkpoint is IFN-γ inducible (35), thus we focused on this as a potential mechanism for the reversal of positive IRE effects. In addition to the increased expression of IFN-γ, we also evaluated protein levels in the blood sera on days 0 – 28 following IRE (**Figure 6A**). IFN-γ levels increased by Day 3 and peaked at Day 9 post-IRE, eventually reducing back to baseline levels by day 21 (**Figure 6A**). Pan02 cells do not express high levels of PD-L1 under baseline or unstimulated conditions (**Figure 6B**). Following *in vitro* stimulation of Pan02 cells with IFN-γ, we observed robust PD-L1 production by western blot (**Figure 6B**). This was specific to IFN-γ, as stimulation with TNF and IL-4 did not have similar changes and the effects of IFN-γ were only partially attenuated by IL-10 (**Figure 6B**). In the *in vivo* tumors, PD-L1 protein levels moderately increased at 3 and 6 days post-IRE treatment and peak at day 14 (**Figure 6C**), which is consistent with the anticipated lag between IFN-γ generation and PD-L1 protein production (**Figures 6A, C**). We also evaluated the temporal activation of STAT1 and STAT3, upstream of IFN-γ and PD-L1, using western blot to detect phosphorylated proteins. Levels of pSTAT3 were relatively maintained in the tumor, whereas p-STAT1 levels peak between days 9 - 21 post-IRE treatment (**Figure 6D**), consistent with the changes in IFN-γ (**Figure 6A**). As alluded to throughout this manuscript, other tumor types appear to have more robust systemic anti-tumor immune system activation with robust effects on metastatic burden (8, 9, 18, 19, 22, 36). This includes the 4T1 mouse mammary gland model of breast cancer (8). Of specific relevance to the data shown here, IFN-γ also appears to be significantly upregulated following IRE-based treatment of the 4T1 cells. Thus, we next sought to directly compare the effects of IFN-γ on PD-L1 levels across tumor cell types, including the mouse Pan02 pancreatic cancer cells, mouse 4T1 mammary tumor cells, DLM8 mouse osteosarcoma cells, and a human U251 glioblastoma cell line (**Figure 6E**). IFN-γ treatment did not result in an increase in PD-L1 levels in the DLM8 and U251 cells; whereas, the Pan02 cells demonstrated a significant increase in PD-L1 (**Figure 6E**). The 4T1 mammary tumor cell line did have a small increase in PD-L1 protein that was attenuated compared to the levels observed in the Pan02 cells (**Figure 6E**). Taken together our data suggest that the IFN-γ produced following IRE treatment in the Pan02 tumors likely contributes to the upregulation of the PD-L1 checkpoint and augments tumor regrowth post-treatment. The robust shift in the pancreatic cancer cells from PD-L1 negative to PD-L1 positive may also explain the attenuated systemic anti-tumor immune response observed in the Pan02 model, compared to other cancers following IRE-based therapy.

## 4 Discussion

IRE and IRE-based therapeutic approaches have shown promise in addressing the challenges posed by this aggressive and often treatment-resistant cancer. One of the key findings of this study is the ability of IRE to induce proinflammatory cell death in pancreatic cancer cells. This is a crucial aspect of IRE’s mechanism of action. Compared to treatment modalities that primarily rely on thermal or ionizing energy, IRE applies high-voltage microsecond pulses to permeabilize the cell membrane irrevocably, leading to immediate cell death and an increased cell death over time as shown in our LDH study. Deciphering the mechanism of the immune response to IRE in pancreatic cancer begins with determining the type/s of cell death induced by the therapy. IRE has a history of inducing apoptosis to necrosis-like cell death (28–30, 37, 38). Our findings here indicate that IRE also induces pyroptosis, which is an inflammatory form of cell death typically associated with damage associated molecular pattern (DAMP) signaling associated with the innate immune system. The induction of pyroptosis is consistent with the pro-inflammatory cytokine release and generally inflammatory conditions identified in the tumor microenvironment post-IRE treatment. The induction of cell cycle arrest in the G0/G1 phase at the lower IRE electric fields is also a novel finding of the current study. This is classically associated with the induction of apoptosis and would be consistent with apoptosis signaling being detected in cells that receive less energy that reside further from the electrodes in the ablation zone.

In addition to inducing cell death, IRE has displayed significant effects on the biological characteristics of the remaining pancreatic cancer cells outside of the treatment margin in the tumor. Our data suggest that these effects are predicted to include a reduction in key cancer hallmarks, including cell proliferation, metastasis, and chromosomal instability. These changes may account for the clinical efficacy of IRE, as the malignancy of the cancer reduces temporarily following treatment and is effective at extending the progression-free survival for a short period. Furthermore, this highlights a critical timing aspect for potential combination therapies or repeated IRE treatments that could further prolong a patient’s survival. Furthermore, modifications in these pathways may open the door to targeted combination therapies that leverage IRE’s cell membrane permeabilization capability. Ongoing investigations into the combination of IRE with chemotherapeutic agents like gemcitabine, a standard treatment for many pancreatic adenocarcinoma patients, hold promise. This combination has the potential to enhance the penetration of chemotherapy into the tumor mass and the tumor cells themselves, possibly resulting in a synergistic effect that improves overall patient survival (12, 14). In fact, electrochemotherapy with reversible electroporation has already proven effective against advanced melanoma and head and neck cancer (39–43). However, the choice of chemotherapy may also affect patient outcomes; while chemotherapies such as FOLFIRINOX may halt tumor progression, long-term use in stable-disease patients has shown little survival differences between patients receiving IRE compared to gemcitabine combination treatments (12, 44). This may be due to the high toxicity of FOLFIRINOX impacting the overall health of the patient or a currently undefined biological difference between how FOLFIRINOX and gemcitabine impact cancer progression that may provide an advantage to gemcitabine (45–47).

In the context of the tumor microenvironment, this study reveals dynamic changes in immune cell populations following IRE treatment. Pancreatic tumors are notorious for creating an immunosuppressive TME, driven by cell types like myeloid-derived suppressor cells (MDSCs) and regulatory T cells. These immune cells play significant roles in promoting an environment that inhibits pro-inflammatory, anti-tumor immune responses. However, IRE appears to alter this landscape by reducing MDSCs and T regulatory cells responsible, in part, for the immunosuppressive barrier identified in pancreatic tumors. These cells may be decreasing due to the semi-specificity of IRE in cell death induction, as they would be concentrated within the ablation zone and continue to decrease in number over time similar to the increased cell death observed in Pan02 cells. Consistent with the reduced immunosuppression, increased DAMP signaling, and elevated innate immune system activation following IRE treatment, other pro-inflammatory immune cell populations, including neutrophils and cytotoxic lymphocytes, are increased in the TME. While our findings show an exciting temporal change to the immune cell populations at the site of tumor treatment, it is clear that regrowth is a significant concern. The eventual shift back to an immunosuppressive and “immunologically cold” TME is a significant hurdle that must be overcome for effective cancer treatment applications.

The MDSC and T-regulatory cell populations eventually begin to repopulate the tumor area while CD8+ and double-positive T cell populations decline by Day 14 post-treatment, which also coincides with the contralateral tumor reduction and regrowth timelines. This appears to be a vital timepoint for the adaptive immune response. While we see an increase in IFN-γ at earlier timepoints after treatment, which would be consistent with the increase in CD8+ T cells seen in the TME, the immune response is likely suppressed by the increase in PD-L1 expression on the tumor cells. PD-L1 is not commonly expressed in Pan02 cells and similarly in many human pancreatic tumors. Consistent with this expression pattern, immunotherapies involving PD1/PD-L1 inhibitors have thus far been relatively ineffective for pancreatic cancer treatment. This is due to the combination of low PD-L1 expression and the immunosuppressive barrier maintained by MDSCs and T regulatory cells (34, 48). Therefore, the increase of inflammation by IRE clearly has initial effectiveness, but overtime the tumor reverts back to its original state and eventually returns to the immunological “cold” TME (49, 50). Consistent with our mouse model data presented here, a recent clinical trial of IRE on pancreatic cancer also showed an increase in the PD1/PD-L1 dynamic. In this study, a higher population of PD-1 positive CD8+ T-cells were observed following IRE treatment (9). Thus, these combined data clearly indicate that the PD-1/PD-L1 checkpoint must be mitigated for long term IRE success as a therapeutic approach in treating pancreatic cancer. The identification of this checkpoint provides insight into tumor regrowth following IRE and likely other tumor ablation modalities. This is especially true when high levels of IFN-γ are generated. Likewise, the shift of a previously PD-L1 negative tumor to one that is PD-L1 positive, along with the temporal changes in the TME, may allow for improved effectiveness of PD-1 targeted checkpoint inhibitors as a co-therapy option. Indeed, a recent preclinical trial on pancreatic cancer has shown beneficial effects of this combination therapeutic approach (25). While we acknowledge that the flank models shown here may not fully recapitulate many features of *in situ* human pancreatic cancer, we believe these findings provide key insights into biological mechanisms that may significantly impact the success of IRE in clinical applications. It is our hope that the current study provides a better understanding of the temporal nature of the induction of this regulatory pathway and allows for IRE co-therapy optimization.

## Data availability statement

The original contributions presented in the study are included in the article/**Supplementary Material**. Further inquiries can be directed to the corresponding author.

## Ethics statement

The animal study was approved by The Virginia Tech Institutional Animal Care and Use Committee. The study was conducted in accordance with the local legislation and institutional requirements.

## Author contributions

KI: Conceptualization, Data curation, Formal analysis, Investigation, Methodology, Visualization, Writing – original draft, Writing – review & editing. RB: Conceptualization, Data curation, Formal analysis, Investigation, Methodology, Visualization, Writing – original draft, Writing – review & editing. NB-W: Data curation, Investigation, Methodology, Writing – review & editing. MP: Investigation, Methodology, Writing – review & editing. KO: Investigation, Methodology, Writing – review & editing. KA: Investigation, Methodology, Writing – review & editing. NA: Investigation, Methodology, Writing – review & editing. ZS: Investigation, Methodology, Writing – review & editing. PE: Investigation, Methodology, Writing – review & editing. BT: Investigation, Methodology, Writing – review & editing. JMM: Investigation, Methodology, Writing – review & editing. AH-W: Investigation, Methodology, Writing – review & editing. SC-O: Data curation, Investigation, Methodology, Writing – review & editing. RD: Conceptualization, Funding acquisition, Supervision, Writing – review & editing. IA: Conceptualization, Funding acquisition, Project administration, Resources, Supervision, Validation, Writing – original draft, Writing – review & editing.
