# Supplementary material for: Irreversible electroporation promotes a pro-inflammatory tumor microenvironment and anti-tumor immunity in a mouse pancreatic cancer model
Source: Front Immunol. 2024 Apr 22;15:1352821. doi: 10.3389/fimmu.2024.1352821 (PMC11070574; doi:10.3389/fimmu.2024.1352821)
Supplement: Supplementary file 1 [file DataSheet_1.pdf]

Supplementary Material

Irreversible electroporation promotes a pro-inflammatory tumor microenvironment and anti-tumor immunity in mouse pancreatic cancer model

Khan Mohammad Imran[1†], Rebecca M. Brock[1†], Natalie Beitel-White[2,3], Manali Powar[1], Katie Orr[4], Kenneth N. Aycock[3], Nastaran Alinezhadbalalami[3], Zaid S. Salameh[3], Paige Eversole[5], Benjamin Tintera[6], Justin Markov Madanick[4], Alissa Hendricks-Wenger[1], Sheryl Coutermarsh-Ott[4], Rafael V. Davalos[3], Irving C. Allen[1,4,7*]

* Correspondence:
Dr. Irving Coy Allen, Department of Biomedical Sciences and Pathobiology, Virginia Tech, Virginia Maryland College of Veterinary Medicine, 295 Duckpond Drive, Blacksburg, VA 24061, USA.
E-mail address: icallen@vt.edu

Supplemental Table 1

Cell Type	Markers
Neutrophils	Cd45+Cd11c-Ly6C-Ly6G+
Mononuclear myeloid-derived suppressor cells	Cd45+Cd11c-Ly6C+Ly6G-
Dendritic Cells	Cd45+Cd11c+F4/80-
Macrophages	Cd45+Cd11c+Ly6C-F4/80+
Cytotoxic Lymphocytes	Cd45+Cd3+Cd4-Cd8+
Double-Positive T cells	Cd45+Cd3+Cd4+Cd8+
T-helper cells	Cd45+Cd3+Cd4+Cd8-
T-regulatory Cells	Cd45+Cd3+Cd4+Cd8-FoxP3+

Supplemental Table 1: Flow cytometry markers used different immune cell types.

Supplemental Figure 1

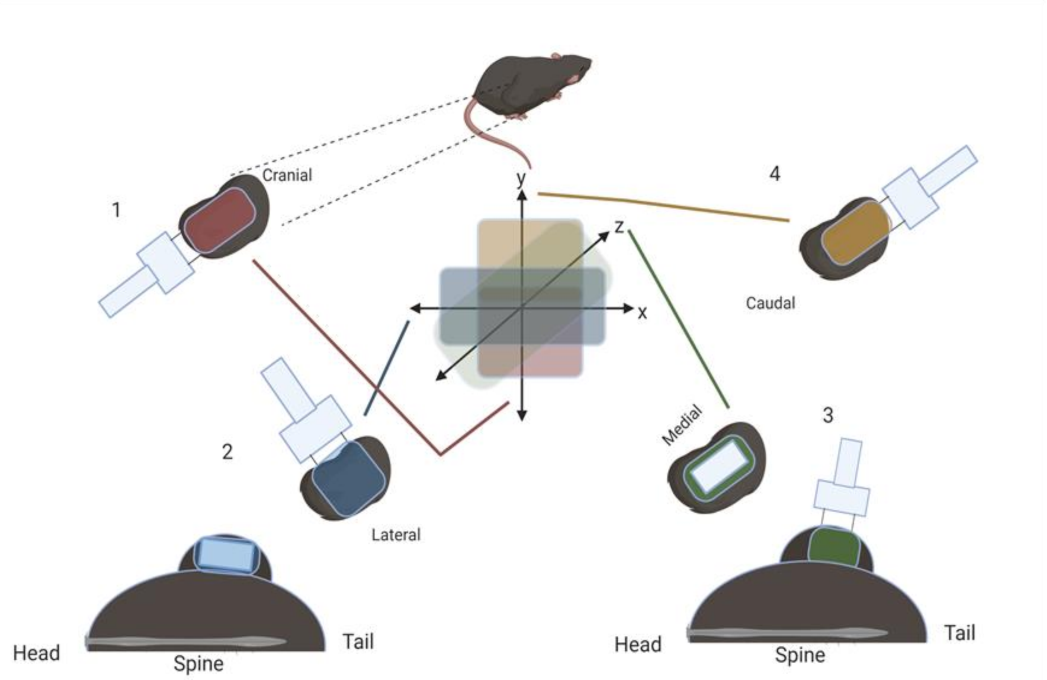

Supplemental Figure 1: Schematic of multidirectional IRE application in mouse flank model. Electrode needles were approximately 21 gauge and spaced 4-5mm apart. Needles were coated to avoid potential arching, leaving exposure at the tips of 5mm.

Supplemental Figure 2

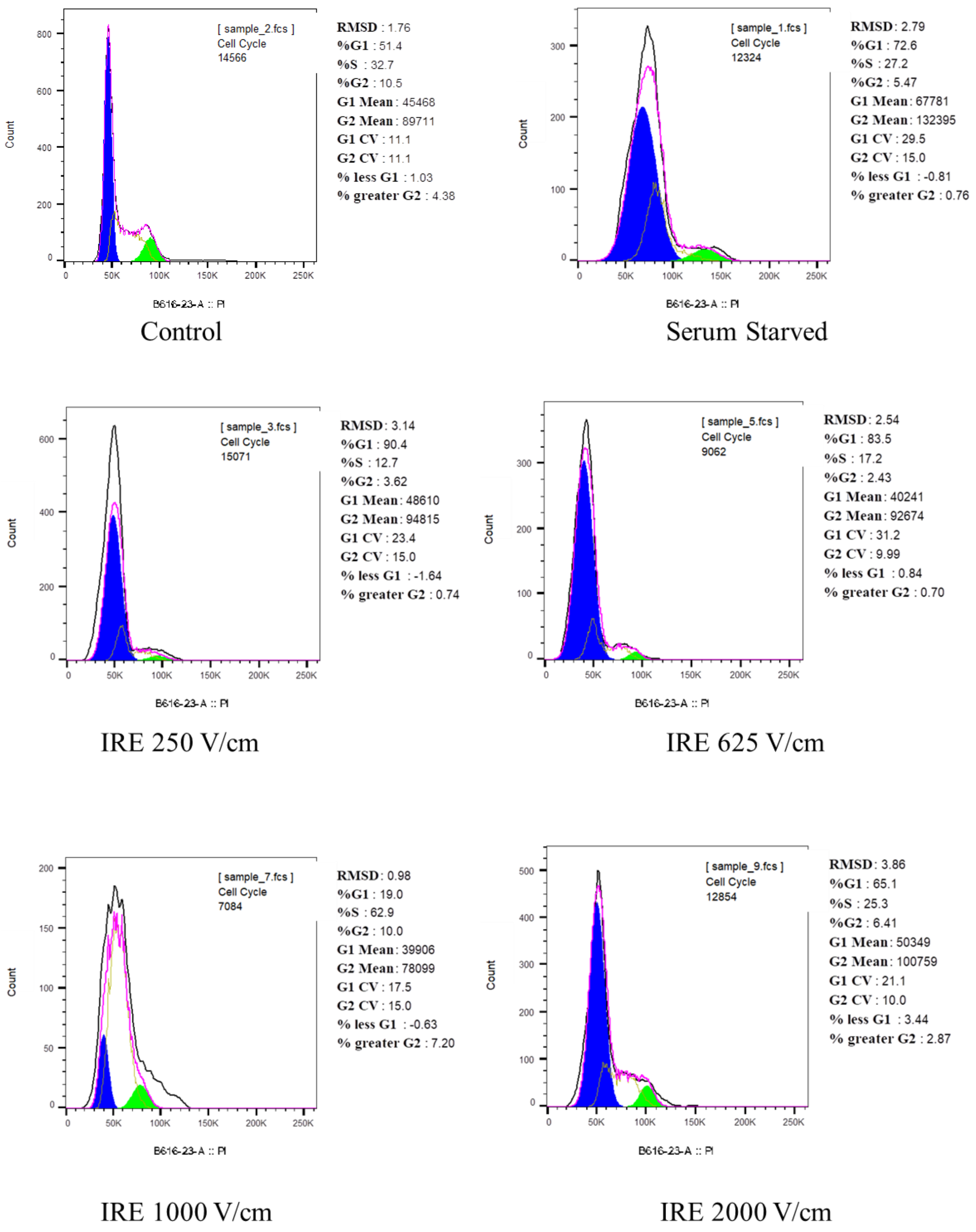

Supplemental Figure 2: Representative flow histogram of cell cycle arrest analysis. Pan02 cells were treated with different voltage of IRE and analyzed with flow cytometry. Serum starved cells were used for positive control since cells get stuck to G0/G1 phase when serum starved.

Supplemental Figure 3

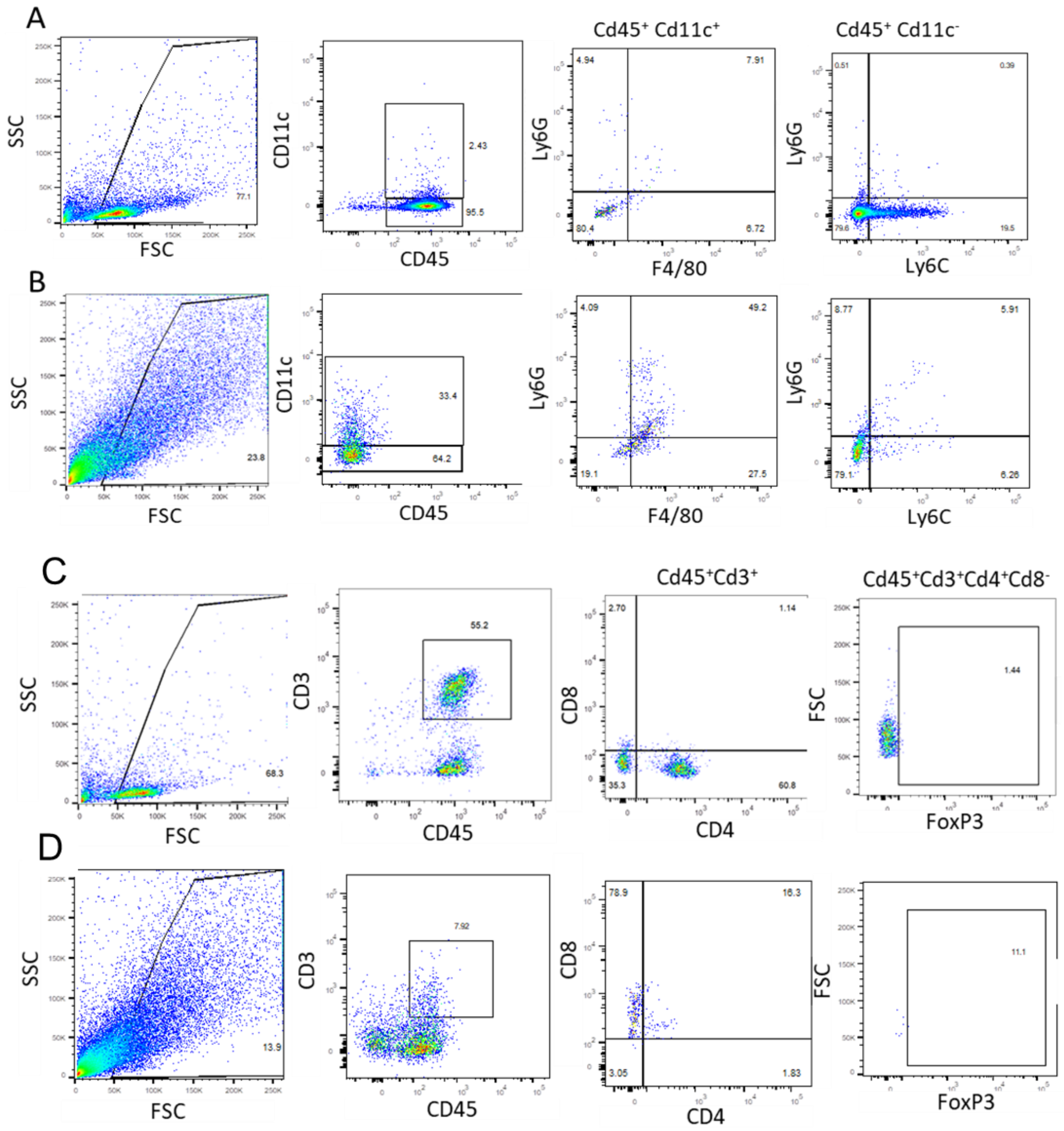

Supplemental Figure 3: Immune cells shift over time post-IRE treatment. Tumors were collected at different time points pre- and post-IRE treatment and cell populations were assessed by flow cytometry. Representative flow plots are shown here as examples of data form untreated (A) and IRE treated (B) tumors, 24hr after treatment. Similar examples of flow plots from lymphocytes are shown for untreated (C) and treated with IRE (D) tumors, 24hr after IRE administration.
